# Multiple Indolent Asymptomatic Yellow-Orange Patches and Plaques

**DOI:** 10.7759/cureus.21870

**Published:** 2022-02-03

**Authors:** Andrew A Niemann, Jason Mammino, Rajiv Nathoo, Robin Burger

**Affiliations:** 1 Dermatology, Kansas City University of Medicine and Biosciences, Kansas City, USA; 2 Dermatology, Kansas City University-Graduate Medical Education Consortium/Advanced Dermatology and Cosmetic Surgery (ADCS) Orlando Dermatology Residency Program, Orlando, USA; 3 Dermatopathology, Advanced Dermatology and Cosmetic Surgery, Delray Beach, USA

**Keywords:** disseminated primary localized cutaneous nodular amyloidosis, primary cutaneous amyloidosis, dermatology, nodular amyloidosis, amyloidosis

## Abstract

An 83-year-old Caucasian male presented with a history of asymptomatic yellow-orange macules and plaques concentrated on his trunk and proximal extremities that have been slowly progressing for the past three years. A punch biopsy revealed the presence of eosinophilic amorphous and fissured material within the superficial and interstitial dermis consistent with nodular amyloidosis. With the lack of concurrent systemic symptoms and negative systemic laboratory workup, the patient was diagnosed with disseminated primary localized cutaneous nodular amyloidosis (PLCNA). Due to the possibility of developing systemic progression, serial monitoring was recommended. This case highlights an under-reported and unusual presentation of a widely distributed form of PLCNA compared to the more common localized nodular and plaque variants.

## Introduction

There are different subtypes of primary cutaneous amyloidosis (PCA), which include: macular, lichen, and nodular [1]. Primary localized cutaneous nodular amyloidosis (PLCNA) is the rarest form of cutaneous amyloidosis where the dermis, subcutis, and blood vessel walls are diffusely infiltrated with amyloid [2]. While the precise pathogenesis of PCA is not well understood, it is believed nodular amyloidosis consists of immunoglobulin ƴ light chains and β2-microglobulin produced by nearby plasma cells [3]. We present a unique case that highlights an unusual widespread distribution of cutaneous localized nodular amyloidosis compared to the more common localized nodular and plaque variants.

## Case presentation

**Figure 1:**
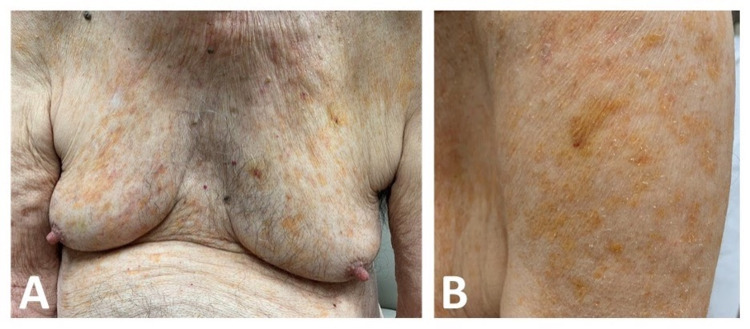
(A) Scattered yellow-orange-hued non-scaly patches with subtle areas of superimposed purpura to the chest and upper abdomen. (B) Similar colored patches and plaques were observed on the left upper outer arm in a subtle reticulated appearance.

An 83-year-old Caucasian male with a past medical history of hypertension, hyperlipidemia, gastroesophageal reflux disease, and two facial basal cell carcinomas presented with asymptomatic, scattered yellow-orange-hued non-scaly macules and thin plaques located on the chest, upper arms, and back coalescing into a reticular pattern (Figures 1A-1B).

He reported the lesions began on his chest and gradually spread to cover a significant portion of his trunk and proximal extremities over the past three years. The patient denies any history of illicit substance use and has a family history of breast cancer in his sister. The patient recalls no preceding trauma and his age-appropriate cancer screenings are up to date. 

Complete blood counts, as well as a comprehensive metabolic panel, erythrocyte sedimentation rate, and urinalysis, were all within acceptable ranges. Serum protein electrophoresis, serum immunofixation, and urine protein electrophoresis were also within normal limits. 

**Figure 2:**
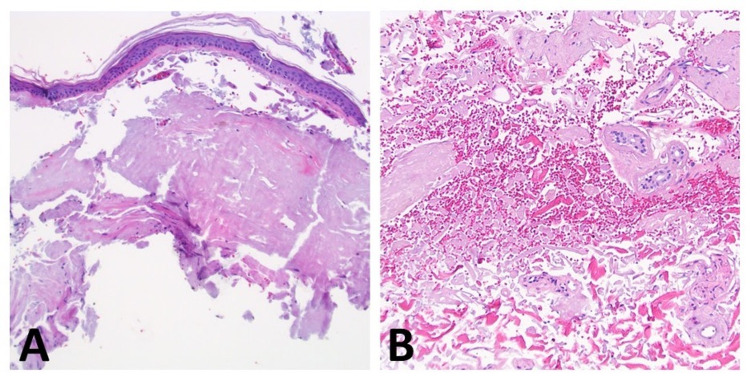
Hematoxylin and eosin staining showing eosinophilic material within the (A) superficial dermis, (B) perivascular, and interstitial regions. (A) 100x magnification; (B) 200x magnification.

Histopathology from two separate skin biopsies showed a homogenous collection of eosinophilic material within the superficial dermis along with a similar appearing substance encompassing dermal blood vessels and surrounding interstitium (Figures 2A-2B). 

**Figure 3:**
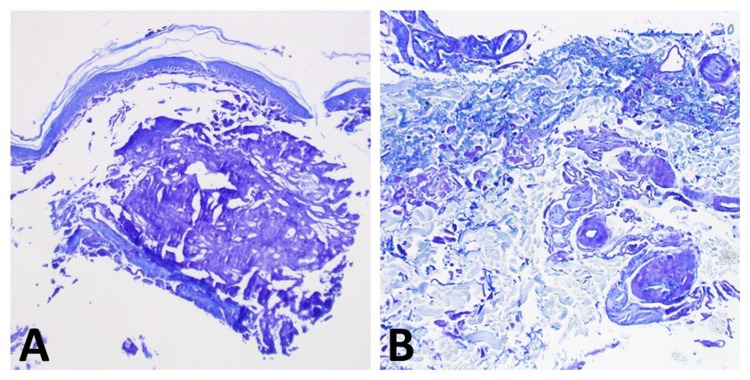
Crystal violet staining highlighting the purple amorphous and fissured material in the (A) superficial dermis, (B) blood vessel walls, and interstitium. (A) 100x magnification; (B) 200x magnification.

The diagnosis of disseminated primary localized cutaneous nodular amyloidosis (DPLCNA) was confirmed with crystal violet staining which further accentuated the purple amorphous material (Figures 3A-3B). Since there is no standard of care for patients diagnosed with DPLCNA, surveillance on a semi-annual basis for signs of morphologic or laboratory progression was advised. 

## Discussion

There are different subtypes of primary cutaneous amyloidosis (PCA), which include: macular, lichen, and nodular amyloidosis [1]. PCA is characterized by extracellular deposition of fibrillar proteinaceous material in the skin without systemic involvement. PLCNA is the rarest form of cutaneous amyloidosis where the dermis, subcutis, and blood vessel walls are diffusely infiltrated with amyloid [2]. 

The precise pathogenesis of PCA is not well understood. Cutaneous macular and lichen amyloidosis originate from degenerated keratinocyte intermediate filaments while nodular amyloidosis consists of immunoglobulin ƴ light chains and β2-microglobulin, which are thought to be produced by plasma cells in the region of the cutaneous deposits [3].

Clinically, PLCNA presents as a single or multiple yellow-brown waxy nodules or plaques with well-defined borders. These plaques may occur focally and occur most commonly on the trunk and extremities with subtle areas of purpura [4]. Highlighted by our case, PLCNA can also present as yellow-orange coalescing reticulated macules and thin plaques in a disseminated distribution. The clinical differential diagnoses include cutaneous lymphoma, leukemia cutis, pseudolymphoma, sarcoidosis, and a xanthomatous process. There is no clear gender predominance, and the age of presentation has ranged from 20 to 87 years, with the 6th decade of life being the most common [5]. Some cases may be associated with Sjogren syndrome, CREST syndrome (calcinosis, Raynaud phenomenon, esophageal dysmotility, sclerodactyly, and telangiectasia), dermatomyositis, and diabetes mellitus [4].

Routine histopathology of PLCNA with hematoxylin and eosin staining typically presents with a pale pink, sometimes fissured, amorphous material in the superficial and deep dermis which can sometimes be found amongst dermal blood vessels and surrounding interstitium [4-5]. Within the infiltrate one can typically find prominent plasma cells [4-5]. Congo red, thioflavin T, pagoda red, and crystal violet staining can all be utilized to highlight the material [5].

Treatment for PLCNA is only required pending the development of systemic manifestations. If the lesions are cosmetically disfiguring or symptomatic, they can be treated via surgical excision, resurfacing lasers, or other destructive methods [2]. All patients with PLCNA should have a systemic evaluation and undergo long-term clinical follow-up to help identify progression to systemic amyloidosis or plasma cell dyscrasias [4]. Fatigue, weight loss, paresthesia, dyspnea, and syncopal attacks due to orthostatic hypertension are all symptoms of systemic progression. Extracutaneous findings associated with systemic amyloidosis include macroglossia, carpal tunnel, and restrictive cardiomyopathy; all of which were not observed in our patient. Fortunately, long-term follow-up of patients with PLCNA has demonstrated that this localized disease has only a 7% progression to systemic amyloidosis [1-2,4,6].

## Conclusions

We present an under-reported subtype of primary cutaneous nodular amyloidosis with a very distinct disseminated appearance. Currently, there is no standard of care for patients diagnosed with DPLCNA. Due to the innumerable lesions, a destructive method was not reasonable or desired by our patient. With a lack of systemic manifestations, treatment with active surveillance on a semi-annual basis was advised. To monitor for systemic progression, we propose a semiannual physical evaluation for abrupt disease morphologic progression with assessment for macroglossia, carpal tunnel, or signs of restrictive cardiomyopathy. In addition, we suggest a yearly laboratory evaluation to monitor for alterations in his complete blood counts, comprehensive metabolic panel, urinalysis, serum and urine protein electrophoresis, and serum immunofixation. Further research should be conducted due to the rarity of DPLCNA, the lack of knowledge known about this variant, and potential differences in progression to systemic disease.
